# Reversing activity of cancer associated fibroblast for staged glycolipid micelles against internal breast tumor cells

**DOI:** 10.7150/thno.36334

**Published:** 2019-09-19

**Authors:** Yun Zhu, Fangying Yu, Yanan Tan, Yun Hong, Tingting Meng, Yupeng Liu, Suhuan Dai, Guoxi Qiu, Hong Yuan, Fuqiang Hu

**Affiliations:** 1 College of Pharmaceutical Science, Zhejiang University, 866 Yuhangtang Road, Hangzhou 310058, People's Republic of China.; 2 Ocean College, Zhejiang University, 1 Zheda Road, Zhoushan 316021, People's Republic of China.; 3 The First Affiliated Hospital, College of Medicine, Zhejiang University, 79 Qingchun Road, Hangzhou 310058, China

**Keywords:** cancer associated fibroblasts, stroma, glycolipid micelles, doxorubicin, telmisartan.

## Abstract

**Rationale**: Nano-carrier based combinational therapies for tumor cells hold great potential to improve the outcomes of patients. However, cancer associated fibroblasts (CAFs) in desmoplastic tumors and the derived pathological tumor stroma severely impede the access and sensitibity of tumor cells to antitumor therapies.

**Methods:** Glycolipid-based polymeric micelles (GLPM) were developed to encapsulate an angiotensin II receptor I inhibitor (telmisartan, Tel) and a cytotoxic drug (doxorubicin, DOX) respectively, which could exert combinational antitumor efficacy by reprogramming tumor microenvironment to expose the vulnerability of internal tumor cells.

**Results:** As demonstrated, *α*-SMA positive CAFs significantly decreased after the pre-administration of GLPM/Tel *in vitro*, which accordingly inhibited the secretion of the CAFs derived stroma. The tumor vessels were further decompressed as a result of the alleviated solid stress inside the tumor masses, which promoted more intratumoral drug delivery and penetration. Ultimately, staged administration of the combined GLPM/Tel and GLPM/DOX at the screened molar ratio not only inhibited the stroma continuously, but also achieved a synergistic antitumor effect through the apoptosis-related peroxisome proliferator-activated receptor-gamma (PPAR-*γ*) pathway.

**Conclusion:** In summary, the strategy of suppressing tumor stroma for subsequent combinational therapies against internal breast tumor cells could provide avenues for management of intractable desmoplastic tumors.

## Introduction

Current trends in clinical research have gradually undergone a shift from a focus on monotherapy to combinational therapies, owing to the complexity, adaptation, and heterogeneity of tumors 1, 2. Specifically, encouraged by the flourishing development of nanotechnology, nano-carrier based formulations have been explored to enhance the robustness and effectiveness of antitumor treatment 3. However, the targets of most combinational therapies are malignant cancer cells, while the interplay of tumor cells and the surrounding tumor stroma on pathology have been largely ignored 4, which severely impedes the outcomes of antitumor therapies.

The tumor stroma, mainly comprised of stromal cells such as CAFs and the derived stromal components, represents the majority of the pathologic structure in desmoplastic tumors such as breast tumor and pancreatic ductal adenocarcinoma 5-8. Notably, tumor vessels are normally embedded into the tumor stroma, in which case the stroma becomes the first and main obstacle for the retention of tumor cells targeting nano-carriers after diffusion from vessels 9. In addition, a well-organized stromal network could form “finger-like” projections that divide tumor masses into distinct compartments, to confine drugs to a limited space so that certain tumor regions are capable of regeneration 10. Furthermore, increased solid stress as a result of proliferative tumor cells, stromal cells and compact stromal deposition compresses perfused vessels, restricting the access of drugs to parts of tumors 11. Accordingly, CAFs are increasingly considered as the primary noncancerous target for antitumor therapy, rather than a bystander 12. For instance, a previous work reported on micelles that are able to target the angiotensin II type I receptor (AT_1_R) that is overexpressed in both CAFs and breast tumor cells 13. However, categories of specific receptors overexpressed on both CAFs and tumor cells are rare, in which case the strategy of restraining the tumor stroma by reversing CAFs activity might be an alternative approach for enhanced antitumor therapy 14, 15.

Telmisartan (Tel), an angiotensin II type 1 receptor blockers (ARBs), inhibits stromal components such as collagen I and HA by decreasing *α*-SMA positive CAFs through transforming growth factor (TGF-*β*) 16-18. The existing matrix could also be destabilized by downregulation of connective tissue growth factor (CTGF) 19, 20. In addition, the structural resemblance between Tel and the peroxisome proliferator-activated receptor-gamma (PPAR-γ) ligand pioglitazone makes Tel a potential agonist of PPAR-γ receptor against tumor cells 21-23, in which case apoptosis events could be enhanced with antitumor chemotherapy-drugs synergistically 24.

**Figure 1:**
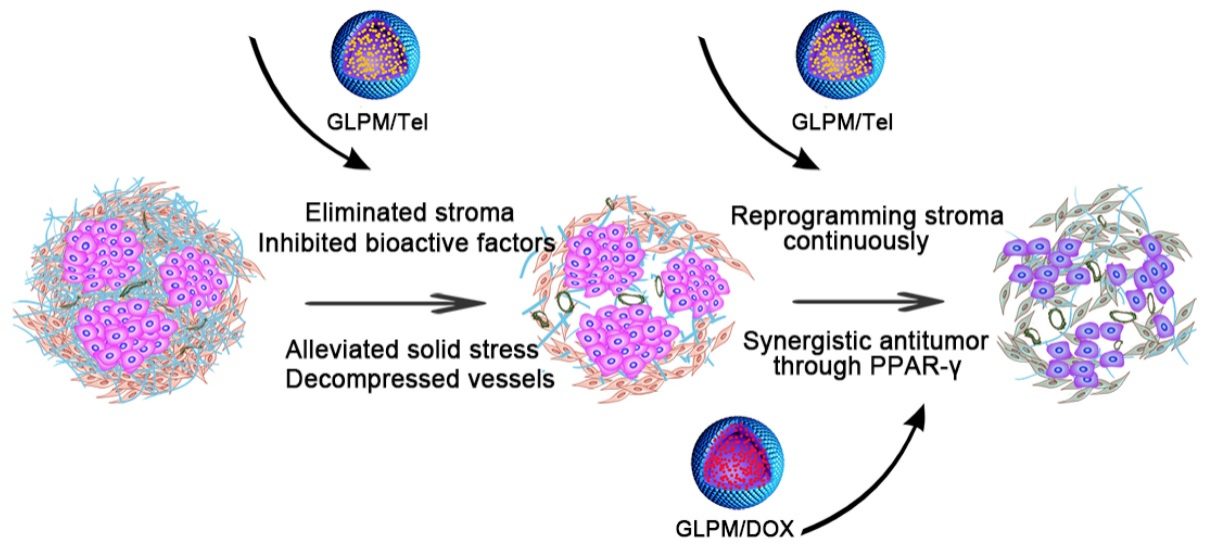
Schematic illustration of the mechanism of remodeling the tumor microenvironment for enhanced synergistic antitumor therapy against breast tumor cells. Pre-administered GLPM/Tel primarily decreased the tumor stroma by reversing the activity of CAFs to alleviate solid stress and facilitate drug penetration in desmoplastic breast tumors. The subsequent administration of combined GLPM/Tel and GLPM/DOX enhanced antitumor efficacy collaboratively through the PPAR-*γ* signal pathway.

To date, the importance of drug order and timing to maximize the synergistic efficacy of combinational nano-therapies against stroma and tumors has not been explored. Herein, an innovative combinational strategy that inhibits the CAFs-derived tumor stroma for the staged administration of combinational therapy against internal breast tumor cells was proposed (Figure 1). In brief, glycolipid polymer-based micelles, composed of biocompatible chitosan (Mw=18.8 k*Da*) and stearic acid were prepared with simplicity and versatility. The GLPM could deliver an ARBs (Tel) into the tumor stroma in virtue of the tumor's pathological structure. Briefly, owing to the decreased activity of CAFs, the pre-administration of GLPM/Tel decompressed tumor vessels as a result of the alleviated solid stress and improved the homogeneity and depth of drug distribution, which jointly improved the efficacy of subsequently administered drugs with different pharmacological actions *in vivo*. In addition, considering the transformation and recruitment of CAFs by cytokines, such as TGF-*β* 14, it is prudent to utilize GLPM/Tel to initiate a tumor microenvironment intervention during the entire antitumor treatment schedule. Thus, after injection of GLPM/Tel, the subsequently delivered combinational therapies (GLPM/Tel and GLPM/DOX at the optimized ratio) were administered not only for synergetic antitumor efficacy in tumor cells, but also for maintenance of the suppressant CAFs derived tumor stroma components.

## Materials and methods

### Materials

Chitosan (Mw=450*k*Da, Yuhuan, China). The stearic acid (SA) was purchased from Shanghai Chemical Reagent Co., Ltd. Both 1-ethyl-3-(3-dimethyl-aminopropyl) carbodiimide (EDC) were purchased from Shanghai Medpep Co, Ltd. Doxorubicin hydrochlorate (DOX.HCl) was obtained from Dalian Meilun Biotech Co., Ltd. Telmisartan and 3-(4, 5-dimethylthiazol-2-yl)-2, 5-diphenyltetrazolium bromide (MTT) were acquired from Sigma-Aldrich Inc. (St Louis, MO). Indocyanine green (ICG) was purchased from Tokyo Chemical Industry (Tokyo, Japan). Trypsin and Dulbecco's modified Eagle's medium (DMEM) were purchased from Gibco-BRL Life Technologies (Carlsbad, CA). Fetal calf serum was purchased from Sijiqing Biology Engineering Materials Co, Ltd (Zhejiang, China). Primary antibodies for *α*-SMA, TGF-*β*, CD31, CCL2, PPAR-*γ*, C-Caspase, HIF-*α* and HABP were purchased from Proteintech Group, Inc. CCL2 primary antibodies was purchased from Wuhan Servicebio technology co., LTD. Other chemicals used were of chromatographic grade or analytical grade.

### Cells and animals

Human breast cancer cell line MCF-7, normal fibroblast NIH/3T3, HUVEC were purchased from the Cell Bank of Shanghai Institute of Biochemistry and Cell Biology, Chinese Academy of Sciences (Shanghai, China). Cells were cultured in DMEM supplemented with 10000U mL^-1^ streptomycin and penicillin respective, 10% fetal calf serum (v/v) at 37 ℃ in an incubator with 5% CO_2_. 5-6 weeks female BALB/c nude mice were purchased from laboratory animal center of Zhejiang University and raised in SPF cleaning room at standard condition. All the researches were carried out based on the guidelines issued by the Ethical Committee of Zhejiang University.

### Synthesis of glycolipid polymer

Chitosan at molecular weight of 18.8*k*Da was gained by degrading 95% deacetylated chitosan (Mw=450k*Da*, Yuhuan, China) with enzymatic 25. The glycolipid polymer (GLPM) was synthesized on the basis of method as reported before with modification 26. Totally, 0.159 g SA and 0.535 g EDC were mixed into 7.5 mL ethanol for 1h at 60 ℃, 0.3 g chitosan (Mw=18*kDa*) were added into 15 mL water at 60 ℃ for 30 min. Then, the solutions were mixed dropwise and stirred at 60 ℃ for 12h. Subsequently, mixtures were dialyzed against water by dialysis bag method (Mw=7 k*Da*, Spectrum Laboratories, Laguna Hills, CA) for 3 days and freeze-dried. The primary products were purified by ethanol for three times to remove the residual substances. The collected solutions were re-suspended into water and collected by lyophilization. The structure of glycolipid polymers was confirmed by ^1^H NMR spectrometer (AC-80, Bruker Biospin, Germany).

### Prepared and characteristics of DOX and Tel loaded micelles

DOX base (DOX) was acquired by freeze-drying after taking out the HCL from doxorubicin hydrochloride with trimethylamine. DOX loaded GLPM (GLPM/DOX) and Telmisartan loaded micelles (GLPM/Tel) were prepared by dialysis method. In brief, 10%(w/w) 2 mg mL^-1^ DOX and 10mg mL^-1^ Telmisartan dissolved into DMSO were added dropwise to GLPM respectively under stirring for 1h. The mixtures were dialyzed against water overnight and centrifuged to remove residual organic solvent and unloaded drugs. The drug encapsulation efficiency (EE%) and drug loading efficiency (DL%) were calculated using a fluorescence spectrophotometer as reported before 27. The hydrodynamic diameters (DLS) and zeta potential of GLPM, GLPM/DOX and GLPM/Tel were measured in triplicate by Anton Paar Litesizer™ 500. The drugs release profiles of DOX, Tel, GLPM/DOX and GLPM/Tel were evaluated by dialysis membrane methods under the PBS (pH 7.4, 10% fetal bovine serum) at 37℃ 28, 29.

### Evaluation for mechanism of remodeling tumor stroma *in vitro*

To get cancer associated fibroblast phenotype, NIH/3T3 cells were pre-treated with collected filtered supernatant with 0.22*μ*m with sterilized filter membrane from MCF-7 for 7 days to acquire activated fibroblasts. The overexpressed biomarker (*α*-SMA) was stained to confirm the success of phenotypic transformation. The acquired activated NIH/3T3 cells were adopted for the following experiments. 2*μ*g/mL Tel and GLPM/Tel were incubated with activated NIH/3T3 cells for 48h at 37 ℃ with 5% CO_2_. The expressed *α*-SMA was evaluated by immunofluorescent staining and fluorescence images were visualized by confocal microscopy. Western blot assays were adopted to investigate the expression of CTGF and *α*-SMA by Tel and GLPM/Tel.

After decreasing activity of CAFs by Tel and GLPM/Tel, the secreted stroma components such as intracellular hyaluronic acid was evaluated *in vitro* 29. 5×10^4^ cells were seeded into 6-well plates and cultured for 12h, which were treated with 2*μ*g/mL Tel and GLPM/Tel for further incubation. After 48h, supernatant was discarded. Cells were washed with PBS three times, harvested and suspended in 0.5mL PBS. Lysis buffer was used to collect intracellular HA as demonstrated. The supernatant was measured by ELISA kits.

HUVEC tube formation assay was performed *in vitro* as reported with modifications 30. Briefly, 50*μ*L/well matrigelTM was seeded onto 96-well plates. 50*μ*L DMEM medium containing 4×10^5^ cells/mL HUVEC cells and 2*μ*g/mL Tel and GLPM/Tel were added into plates. 6h later, the tube formation was measured and captured by microscope. Cells treated with PBS were served as a negative control.

### *In vitro* cell viability evaluation and analysis of synergistic drug combinations

MTT assays were performed to evaluate *in vitro* viability of Tel, GLPM/Tel against activated NIH/3T3 and MCF-7 cells respectively. Briefly, 2×10^3^ activated NIH/3T3 cells/well were added into 96 well plates and incubated overnight, gradient concentration (2μg/mL, 4μg/mL, 6μg/mL, 8μg/mL) of Tel and GLPM/Tel were cultured for 48h; 2×10^3^ MCF-7 cells/well were added into 96 well plates and incubated overnight, gradient concentration (2.4μg/mL, 6μg/mL, 12μg/mL, 24μg/mL) of Tel and GLPM/Tel were cultured for 48h, 20*μ*L 5mg mL^-1^ MTT was dropped into each well. Cell viability was evaluated by measuring the absorbance at 570 nm with universal Microplate Reader (EL800, BIO-TEK Instruments Inc., USA). The capacity of internalization of Tel and GLPM/Tel by activated NIH/3T3 cells and MCF-7 were explored. 5×10^4^ cells were added into 6-well plates for incubation overnight and 2*μ*g/mL Tel and GLPM/Tel were incubated for 2h and 4h. Residual drugs were removed from plates and cells were washed three times with PBS. Afterwards, 1mL DMSO was added into each well, ultrasonic equipment was used to destroy structure of internalized GLPM/Tel for 60mins. After centrifugation of the solution at 4000 rpm/min for 10 mins, the supernatant was measured by fluorescence spectrophotometer.

To explore the mechanism underlying, 5×10^4^ MCF-7 cells in DMEM supplemented with 10% FBS were seeded into 6-well plates and incubated overnight. PBS, Tel and GLPM/Tel at 2*μ*g/mL were added into each well for the incubation for 48h incubation. Then, antitumor efficiency of Tel and GLPM/Tel through PPAR-*γ* in MCF-7 breast cancer cells* in vitro* was examined by western blot assay 31. Caspase-3 is major member of the cysteine aspartic acid-specific protease (caspase) family that plays key roles in apoptosis in cancer cells 24. The activity of caspase was performed with antibodies against C-caspases3 according to the manufacturer's recommendations.

According to Chou and Talalay methods 32, combination index (CI) calculation of combined Tel and DOX, GLPM/Tel and GLPM/DOX respectively was performed with CompuSyn software on the basis of results of MTT, in which the molar ratio of Tel to DOX, GLPM/Tel to GLPM/DOX was 4 to 0.5. Briefly, for each level of Fa (the fraction of affected cells), the CI values of combined Tel and DOX or GLPM/Tel and GLPM/DOX were collected in virtue of the equation below: CI=(D)_1_/(D_x_)_1_+ (D)_2_/(D_x_)_2_. Specifically, (D)_1_ and (D)_2_ means dosage of each drug in the combination therapy attributing to 100 percentage growth inhibition rate, (D_x_)_1_ and (D_x_)_2_ indicated dosage of single drug attributing to 100 percentage antitumor effect 32. The CI values for drug combinations to Fa were matched to explore underlying patterns. As verified, CI values less than 1 indicated synergism effect while above 1 meant antagonism of combined drugs, respectively. In addition, CI values between Fa 0.2 to 0.8 are normally considered validate 33.

Annexin V/PI assays were adopted to investigate the synergetic antitumor efficacy of combinational GLPM/Tel and GLPM/DOX on MCF-7 cells. Briefly, 5×10^4^ cells were added into 6-well plates overnight. GLPM/Tel (1*μ*g/mL of equivalent Tel), GLPM/DOX (0.5*μ*g/mL of equivalent DOX), GLPM/Tel and GLPM/DOX at equivalent drugs were dropped into plates and incubated at 37℃ overnight. Afterwards, MCF-7 cells were collected for staining by Annexin V/PI as protocol and measured by FACS.

### Penetration and antitumor efficacy evaluation in three-dimensional multicellular tumor spheroids

To evaluate penetration capacity of drugs after decreasing activity of CAFs, the MCTSs, comprising of activated NIH/3T3 and MCF-7 cells, was constructed to stimulate tumor microenvironment *in vitro* by the “handing drop” technique on the basis of methods as reported 13. Briefly, 50*μ*L sterilized agarose solution (1.5%, w/v) was seeded into 96-well plates to form a smooth-faced thin layer after cooling at room temperature for 2h. Then, 180*μ*L DMEM containing 10% fetal bovine serum was dropped into the pre-treated wells later. 10*μ*L mixture containing 1000 activated NIH/3T3 and 1000 MCF-7 cells were added on to the lid of agarose covered 96-well plates.

2*μ*g/mL Tel and GLPM/Tel were incubated with MCTSs for 48h when the diameter of MCTSs get appropriate diameter. Subsequently, GLPM/DOX (containing 0.5*μ*g/mL DOX) was added into the pre-treated MCTSs for further 24h incubation. The MCTSs were collected, washed with PBS and fixed by 4% formaldehyde at room temperature. Lastly, the Z-stack images of penetrated MCTSs were observed by confocal microscopy. For evaluation of antitumor efficacy, five groups (Control, GLPM/Tel, GLPM/DOX, GLPM/Tel+GLPM/DOX and GLPM/Tel+combinational therapy) were divided randomly and administered respectively. 2*μ*g/mL GLPM/Tel was incubated in second group, forth group and fifth group for 36h when the diameter of MCTSs get approximate 200*μ*m in GLPM/Tel (1*μ*g/mL) groups. Afterwards, the culture medium is replaced with fresh medium and GLPM/DOX is added into third group and fourth group, GLPM/Tel and GLPM/DOX at molar ratio of 2:1 was added into fifth group for further 36h incubation. The treated cells are collected and stained by propidium iodide (PI) by FACS.

### Tumor masses specific accumulation evaluation *in vivo* after GLPM/Tel treatment

5×10^6^ MCF-7 cells suspended in 100 mL DMEM without fetal calf serum were injected into the flank of nude mice for subsequent experiments *in vivo*. When tumor volume reached 100mm^3^, PBS, Tel and GLPM at the concentration of 200*μ*g/mL was intravenously administered three times every other day. Afterwards, ICG labelled GLPM was subsequently injected into veins of the tails to explore the changed deposition in tumor masses. The mice were visualized by Maestro *In vivo* Imaging System (CRI Inc., Woburn, MA) at 36h post-injection and sacrificed for excised tumor tissues. The fluorescence intensity of excised tumor masses was imaged and calculated by imageJ software. The changing distribution of ICG labelled GLPM in tumor masses after drugs treatment was investigated by confocal microscopy.

To verify the efficacy of remodeling tumor stroma by Tel and GLPM/Tel, red fluorescence DiI loaded GLPM (GLPM/DiI) was administered intravenously after three times injection of GLPM/Tel to demonstrate the correlation of CAFs, tumor vessels and penetrated GLPM/DiI. After distribution of GLPM/DiI for 36h, tumor masses were excised and dissected for staining of *α*-SMA, CD31 by immunofluorescent staining. The fluorescence images were collected by confocal microscopy. The underlying correlation of CAFs, CD31 and DiI were counted up by imageJ software. The volume of tumor and weight of breast tumor bearing mice was also monitored during the treatment. The weight of excised tumor masses was also measured at the end of drugs treatment.

When the volume of tumor reached about 150mm^3^, the animals were administered with PBS, Tel and GLPM/Tel for three times every other day. After drugs treatment, tumor masses were excised and washed with PBS. To measure solid stress, the tumor masses were cut along its longest axis (∼80% of its thickness). Subsequently, the tumor masses were relaxed for about 10 min. Then, the opening at the surface of the tumor was measured 34.

After administration of Tel and GLPM/Tel, masson staining was used to visualize changes of collagen fibers in tumor masses. Hyaluronic acid was stained by immunohistochemical staining method by the HABP primary antibody. The bioactive substance TGF-*β* and chemokine 2 (CCL2) was evaluated by immunohistochemical staining to evaluate efficiency of remodeling tumor stroma by Tel and GLPM/Tel. Hypoxia-inducible factor α (HIF-α) was stained by immunofluorescent staining. All stained slides were visualized under microscope randomly.

### *In vivo* antitumor efficacy and safety evaluation

Breast tumor-bearing nude mice were divided into 8 groups for antitumor efficiency evaluation: 1) PBS; 2) Tel; 3) DOX. HCL; 4) Tel+ combined therapy (Tel and DOX at molar ratio of 2:1); 5) GLPM/Tel; 6) GLPM/DOX; 7) GLPM/Tel+GLPM/DOX; 8) GLPM/Tel+combination (GLPM/Tel and GLPM/DOX at molar ratio of 2:1). The total equivalent pre-treated 3 mg kg^-1^ Tel were administered via tail vein when volume of tumor reached approximately 150mm^3^, the DOX at 3 mg kg^-1^ was subsequently administered. Briefly, Tel was pre-administered into groups of 2) and 4). The same dosage of GLPM/Tel was injected into groups of 5), 7) and 8) for one week every other day. Subsequently, DOX, combined Tel and DOX, GLPM/DOX and combined therapy (GLPM/Tel and GLPM/DOX) were further administered intravenously for the evaluation of antitumor therapy. The tumor volume and body weight were monitored every two days. At the end of treatment, tumor masses were excised for weighing the weight. The apoptosis profiles of tumor cells were detected with HE staining (hematoxylin and eosin stain) and TUNEL kits to confirm the antitumor efficiency of different administration strategy. In brief, tumor masses were fixed into in 4% paraformaldehyde solution dissolved in PBS and embedded into paraffin to get dissected sections at thickness of 5*μ*m. HE staining and TUNEL kits was used and the sections were observed by optical microscope to confirm the antitumor therapy of purposed strategy. In addition, weight of breast tumor bearing nude mice was also monitored and HE staining was adopted for major organs after different drugs treatment to measure the safety after drugs treatment.

### Statistical Analysis

Results were presented as mean ± standard derivation. The analysis of difference was examined by two-tailed student's t-test. A p value of p < 0.05 was considered statistically significant.

## Results and Discussion

### Preparation and characterization of drug-loaded micelles

**Figure 2:**
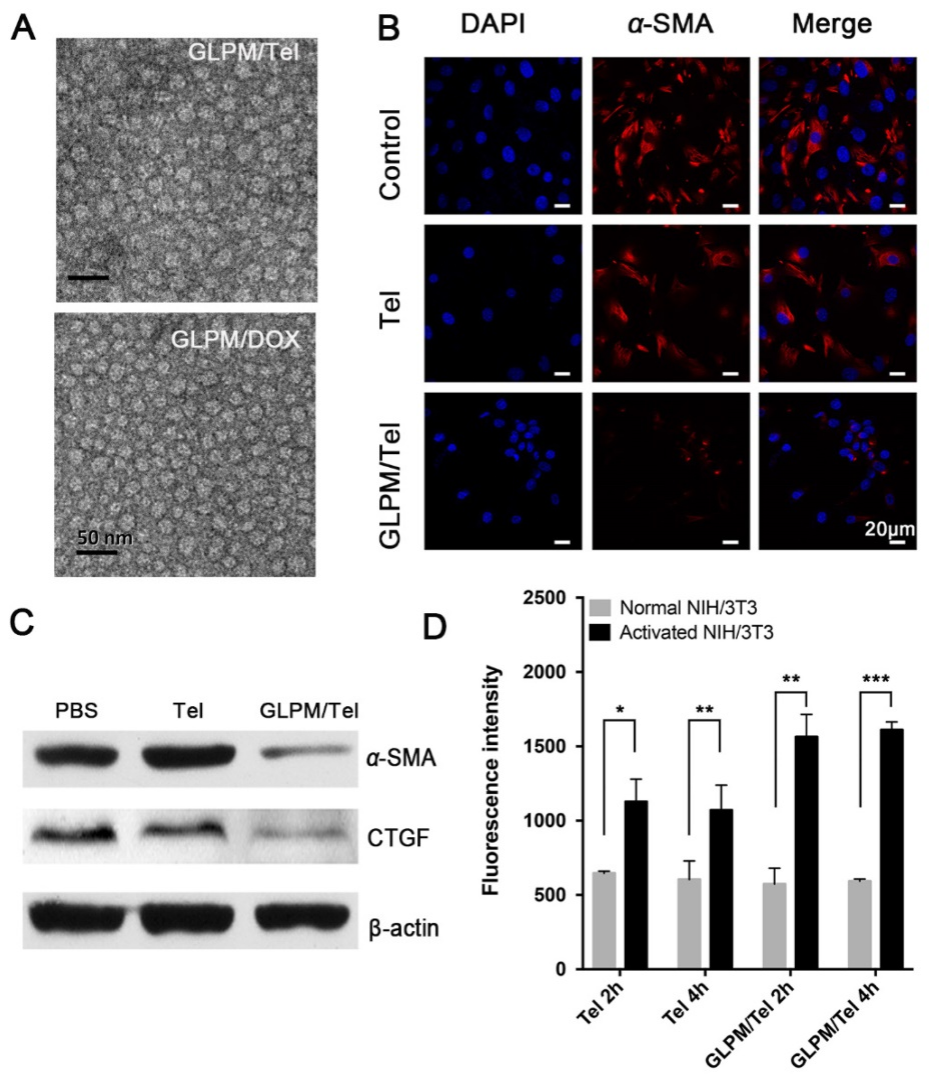
Pharmacological action evaluation of GLPM/Tel in activated NIH/3T3 cells. A) Images of GLPM/Tel and GLPM/DOX visualized by TEM. B) Immunofluorescence staining analysis of α-SMA expression after different drugs treatment for 48h. C) Western blot analysis of the α-SMA and CTGF proteins after PBS, Tel and GLPM/Tel incubation for 48h. D) The intracellular uptake of Tel and GLPM/Tel on normal NIH/3T3 and activated NIH/3T3 cell lines. *p < 0.05, **p < 0.01, ***p < 0.001 as determined by two-tailed student's t-test.

^1^H NMR spectroscopy was utilized as shown in Figure S1. The peaks at 1.3 ppm and 3.6 ppm in GLPM could be attributed to the -CH_3_ group in stearic acid and the protons in chitosan respectively. The critical micelle concentration (CMC) of GLPM was calculated as 45.7 *μ*g/mL as shown in Figure S2. Hydrophobic drugs can interact with hydrophobic core of amphiphilic polymers by “Van der Waals forces” in aqueous medium 35. Thus, GLPM can be utilized to encapsulate hydrophobic Tel and DOX respectively by dialysis to increase stability and prolong circulation time *in vivo*. As shown in Table 1 and Figure S3, Tel and DOX were successfully encapsulated in GLPM with drug loading efficiencies of 8.06% and 8.15%, and encapsulation efficiencies of 87.6% and 88.2%, respectively. The optimized GLPM with 7.1% substituted SA (molar ratio of the sugar ring in the chitosan backbone) was at the size of 138.59±18.68 nm, which was chosen for subsequent research. The zeta potential of GLPM was recorded as 28.7±1.2 mV. After drug loading, the size of GLPM/Tel and GLPM/DOX decreased to 76.32±4.30 nm and 110.65±3.46 nm, which was suitable for tumor accumulation in desmoplastic tumors. The zeta potentials of GLPM/Tel and GLPM/DOX decreased to 26.6±0.7 mV and 23.9±2.0 mV, respectively. The PDI confirmed good monodispersity of drugs loaded micelles. The morphology of the drug loaded micelles was also visualized by transmission electron microscope (TEM) as shown in Figure 2A, which showed the spheroid-like properties and monodispersity.

The drug release kinetics of Tel, GLPM/Tel, DOX and GLPM/DOX were performed by dialysis methods against PBS (pH 7.4, 10% fetal bovine serum) at 37℃ to evaluate the stability of GLPM/Tel and GLPM/DOX *in vitro*. As shown in Figure S4, both DOX and Tel exhibited over 80% burst drug release after 24 h, and almost 100% of DOX and 91.48% of Tel was released after 72h. In contrast, only 35.87% Tel was released from GLPM/Tel and less than 50% DOX was released from GLPM/DOX even after 72 h. Collectively, the synthesized GLPM were superior in reducing premature release of the payload, which could improve the tumor mass specific accumulation and avoid off-target toxicity for normal tissue *in vivo*.

### Evaluation of the mechanism of tumor stroma remodeling *in vitro*

To acquire a cancer associated fibroblast phenotype *in vitro*, normal fibroblast NIH/3T3 cells were first pre-cultured with supernatant collected from MCF-7 cells for one week. The *α*-SMA that was used to identity the CAFs was then stained. As shown in Figure S5, almost all pre-treated NIH/3T3 cells exhibited an *α*-SMA positive phenotype in contrast to normal NIH/3T3 cells, verifying the successful transdifferentiation of the CAFs.

As demonstrated, ARBs can prevent matrix production by decreasing the activity of CAFs, and the expression of *α*-SMA on activated NIH/3T3 cells were evaluated by immunofluorescence staining after treatment with 2*μ*g/mL Tel and GLPM/Tel (equivalent Tel) for 48 h. As shown in Figure 2B, the amount of *α*-SMA decreased after GLPM/Tel treatment. In contrast, Tel could reduce only limited amounts of *α*-SMA. Western blotting was also adopted to evaluate the efficacy of decreasing the amount of activated NIH/3T3 cells (Figure 2C), which was consistent with the results of the fluorescent staining. As calculated by imageJ software in Figure S6 and Figure S7, a total decrease of 81.23% in *α*-SMA was observed after GLPM/Tel treatment, while little effect was observed after Tel treatment. In addition, CTGF, which is downstream of angiotensin signaling, was reported to stabilize transient fibrosis 36. As demonstrated, only 32.01% of CTGF was inhibited by Tel, while GLPM/Tel reduced the expression of CTGF by 82.39%. Collectively, the consistent trend of CTGF and *α*-SMA expression led to the conclusion that GLPM/Tel has the great superiority to Tel when decreasing activated NIH/3T3 cells, which could further show a profound influence on the secretion and stability of tumor stromal components. Considering the original pharmacological activity of decreasing blood pressure, the probable inhibition of capillaries by Tel and GLPM/Tel was also evaluated on human umbilical vein endothelial cells (HUVECs) as shown in Figure S8. Both Tel and GLPM/Tel showed remarkable inhibitory effect on HUVEC tube formation at 2 *μ*g/mL compared with the PBS group, which could prune tortuous and disordered vessels to reduce vessel hyperpermeability for improved blood perfusion *in vivo* 37, 38.

Compared to quiescent, inert and supportive fibroblasts in nonmalignant tissue, CAFs are proliferative, migratory, and highly secretory cells, in which case the cellular behaviors, such as uptake, could also show differential patterns. Thus, the enhanced pharmacological activity of GLPM/Tel might be attributed to improved uptake capacity *in vitro*. The uptake efficiency of Tel and GLPM/Tel by normal NIH/3T3 cells and activated NIH/3T3 cells are shown in Figure 2D. Normal NIH/3T3 cells exhibited a time independent uptake behavior, and there was little difference between the amount of internalized Tel and GLPM/Tel. In contrast, both Tel and GLPM/Tel exhibited a 2-fold increase in uptake by activated NIH/3T3 cells in contrast to that of normal NIH/3T3 cells at 2h and 4h, respectively. Activated NIH/3T3 cells showed a higher capacity to internalize GLPM/Tel compared to normal NIH/3T3 cells, the different internalization capacity of Tel and GLPM/Tel in activated NIH/3T3 cell could be mainly attributed to the different way of uptake 39. Apart from the destabilized matrix after GLPM/Tel treatment, the secretion of intracellular matrix components, such as HA, was also investigated by ELISA kits *in vitro*. As shown in Figure S9, Tel slightly decreased the intracellular HA. In contrast, GLPM/Tel showed a higher inhibition effect of intracellular HA secretion. To exclude the possibility of cytotoxicity from Tel and GLPM/Tel in activated NIH/3T3 cells, MTT assays were performed. As shown in Figure S10, neither Tel nor GLPM/Tel affected cell viability.

### *In vitro* analysis of the synergistic antitumor effects of combinational drugs on MCF-7 cells

**Figure 3:**
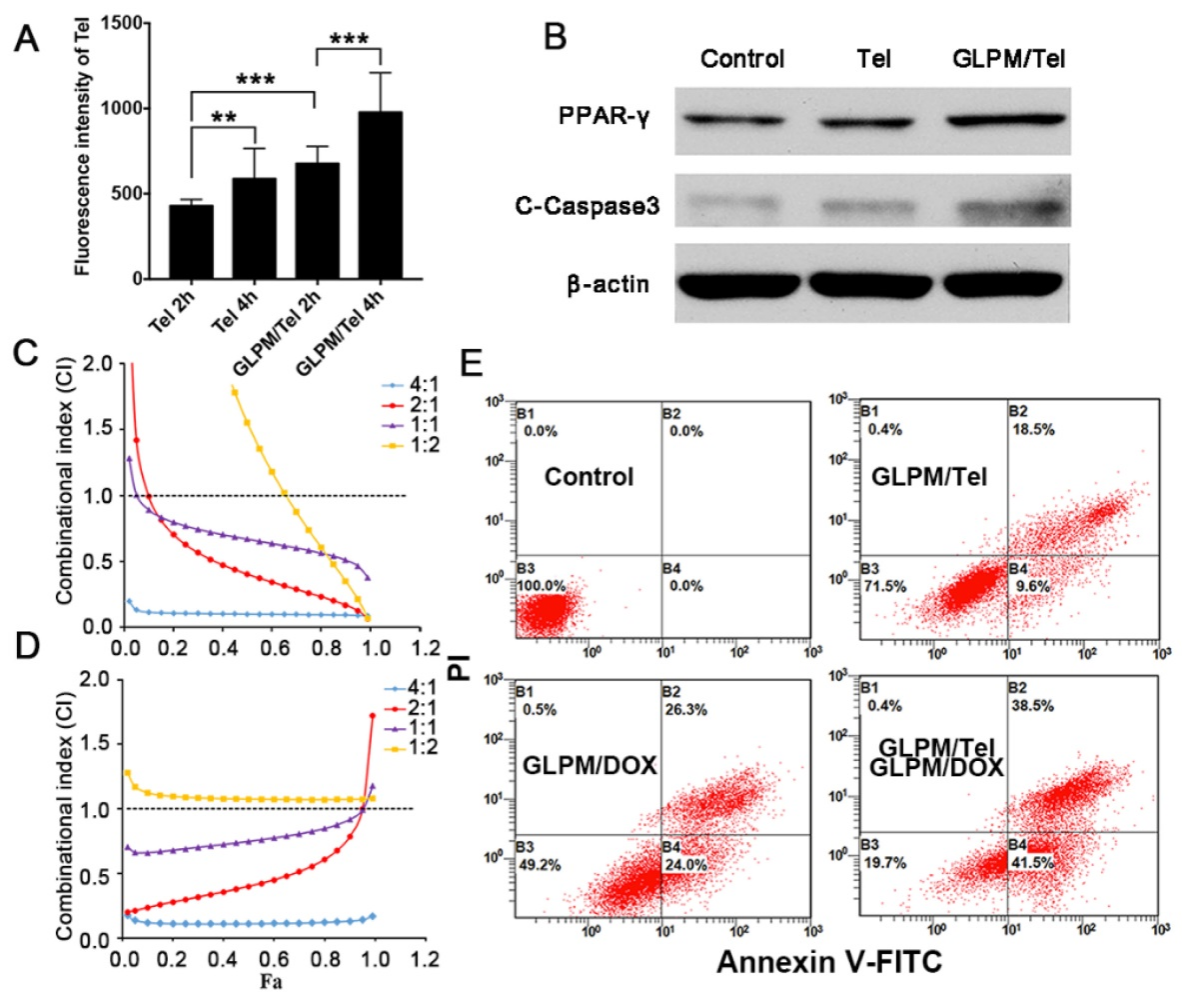
The evaluation of synergistic antitumor efficiency between GLPM/Tel and GLPM/DOX in MCF-7 cells *in vitro*. A) Intracellular uptake of Tel and GLPM/Tel on MCF-7 cells. B) Western blot analysis of apoptosis related PPAR-γ and cleaved-caspase 3 proteins after drugs treatment for 48h. The combination index (CI) vs Fa plots of C) combinational Tel and DOX, D) GLPM/Tel and GLPM/DOX was calculated according to the MTT assays. E) Evaluation of antitumor efficacy by Annexin V-FITC/PI staining after different drugs treatments. **p < 0.01, ***p < 0.001 as determined by two-tailed student's t-test.

Telmisartan, a ligand for PPAR-γ 31, has an anticancer effect through the PPAR-γ dependent pathway in cancer cells 22, in which case we put forward the hypothesis that telmisartan might have synergistic effect with commonly used apoptotic drugs such as DOX. First, the antitumor efficacy of Tel and GLPM/Tel on MCF-7 cells was investigated, as shown in Figure S11. Tel only showed a limited anti-proliferative effect. In contrast, GLPM/Tel exhibited significant cytotoxicity as a result of the rapid and enhanced uptake in MCF-7 cells (Figure 3A). The western blot method was utilized to further confirm the underlying mechanism of enhanced anti-proliferative effect by GLPM/Tel. As shown in Figure 3B, Tel showed little effect on the expression of PPAR-γ and cleaved-caspase 3, which are apoptotic-related proteins. However, obviously increased levels of both proteins were observed after GLPM/Tel treatment in MCF-7 cells.

Subsequently, the antitumor efficacy of a combination of Tel and DOX at different ratios was carried out for 48h on MCF-7 cells *in vitro*. The combination index (CI) was calculated by CompuSyn software according to Chou and Talaly 32. As indicated in Figure 3C, the CI for the combined Tel and DOX at ratios of 4:1, 2:1 and 1:1 all fell between 0.2 and 0.8, which suggested a synergistic effect. The molar ratio of Tel and DOX at 1:2 showed antagonistic effect at low Fa (fraction of affected cells) while exhibiting a synergistic effect at high Fa. In contrast, there were some differences in the groups of combined nano-agents, as shown in Figure 3D. The antagonistic effect could be observed for the combined therapy at a ratio of 1:2 over all ranges of Fa; however, all three other ratio combinations exhibited better synergistic effects compared with the combination of the free drugs. Specifically, the combination of GLPM/Tel and GLPM/DOX at the molar ratio of 2:1 was verified to hold better synergetic efficacy over a range of concentrations. Considering the synergistic effect and the preferred low dosage of administered Tel* in vivo*, the molar ratio of 2:1 for GLPM/Tel and GLPM/DOX was chosen for further experiments. Annexin V/PI assays were utilized to further evaluate the synergistic efficacy of GLPM/Tel and GLPM/DOX* in vitro*. The results in Figure 3E indicate that only 28.5% of MCF-7 cells underwent apoptosis after GLPM/Tel (1 *μ*g/mL of equivalent Tel) treatment for 48h, and a higher cytotoxicity was observed after GLPM/DOX (0.5 *μ*g/mL of equivalent DOX) treatment. In contrast, a super-additive antitumor efficacy of MCF-7 cells at apoptotic proportion of 81.3% was achieved in the group of the combined NPs group, providing evidence of the synergistic efficacy of GLPM/Tel and GLPM/DOX.

### The enhanced distribution of subsequently administered drugs by GLPM/Tel

It has been gradually recognized that breast tumors can be classified as either tumor stroma abundant and low-penetrative tumors^40^, ^41^. An MCF-7 tumor mass was first sliced and stained to visualize the pathological structures by immunofluorescent staining. As shown in Figure S12, the green fluorescence labelled CAFs presented a well-organized pattern and overlapped with the red fluorescence labelled vessels 13, which could lead to restrained drug transport in the dense stroma instead of inside the tumor cells. Moreover, the dense ECM deposition and tumor fibrosis by CAFs compressed the vessels to decrease the delivery of antitumor drugs 11. Thus, decreasing *α*-SMA positive CAFs with GLPM/Tel could improve the drug delivery capacity and permeation capacity through inhibiting of the secretion of stroma components.

**Figure 4:**
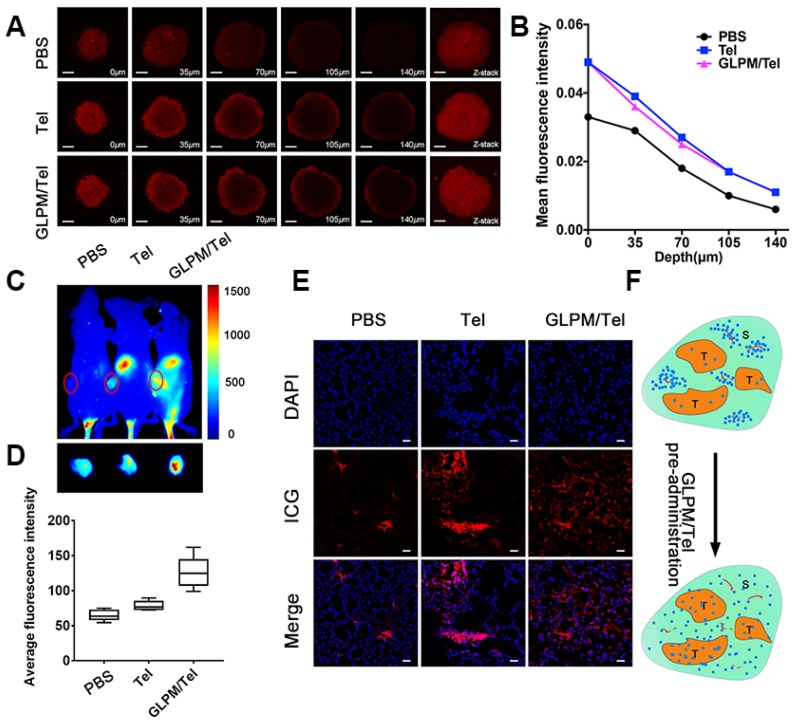
Enhanced drugs penetration by pre-administrated GLPM/Tel *in vitro* and *in vivo*. A) Z-stack images of penetration of GLPM/DOX in MCTSs were visualized by confocal microscopy after PBS, Tel and GLPM/Tel treatment for 2 days. Scale bar: 200*μ*m. B) Curves of fluorescence intensity vs different depth in MCTSs calculated by ImageJ software. C) Fluorescent images of ICG labelled GLPM distribution on breast xenografts at determined time intervals after saline, Tel and GLPM/Tel treatments for three times. The tumor position is circled by the red lines. D) Semi-quantitative analysis of fluorescence intensity from the excised tumor masses. E) Distribution patterns of ICG labelled GLPM *in vivo*. Bar: 50μm. F) Graphic illustration of changed distribution patterns in breast tumors after drug treatments. T: tumor cells area, S: stromal area, red line: blood vessel, blue dots: accumulated drugs.

The pharmacological activity of GLPM/Tel on activated NIH/3T3 cells was verified *in vitro* above, and the improved permeation effect was further investigated. First, multicellular spheroids (MCTSs) comprising activated NIH/3T3 cells and breast tumor cells (MCF-7) at a ratio of 1:1 were constructed 42, 43. The distribution pattern of the mixed cells in our previous works confirmed the applicability for simulating cell distribution *in vivo*. Accordingly, MCTSs were incubated with GLPM/DOX for 24h and visualized by confocal microscopy after pre-treatment with saline, Tel or GLPM/Tel (2 *μ*g/mL) for 48h. As shown in Figure 4A and Table S1, almost no red fluorescence could be observed in PBS group at a thickness of 105 *μ*m. In contrast, significantly improved permeation of GLPM/DOX up to 140 *μ*m could still be visualized after Tel and GLPM/Tel treatment. The semi-quantitative analysis of fluorescence intensity vs depth in MCTSs by ImageJ software in Figure 4B further confirmed the potential of Tel and GLPM/Tel for enhanced drug penetration *in vitro*. Similar result was also observed in MCTSs composed of 4T1 cells and activated cells as shown in Figure S13. The antitumor efficacy was also evaluated by PI staining *in vitro* as shown in Figure S14 and Table S1. Only 13.0% of the cells were apoptotic after GLPM/Tel treatment. GLPM/DOX exhibited the expected higher cytotoxicity of 30.0% apoptotic cells. The administration of GLPM/Tel and GLPM/DOX in sequence showed an increase in cytotoxicity of 37.3% apoptotic cells due to increased drug penetration. In contrast, 53.5% of cells were apoptotic after implementation of the GLPM/Tel+combined NPs strategy (GLPM/Tel and GLPM/DOX at a molar ratio of 2:1), which could be attributed to the enhanced drug penetration and synergistic antitumor effects on tumor cells.

To investigate whether Tel and GLPM/Tel affect the distribution of subsequently administered drugs *in vivo*, the near-infrared fluorescent probe indocyanine green (ICG) was used to label GLPM to monitor the distribution behavior *in vivo* by the Maestro *In vivo* Imaging System at predetermined time points after three pre-treatments of saline, Tel or GLPM/Tel. As shown in Figure 4C, relatively weak fluorescence of ICG labelled GLPM could be visualized at the tumor site of the saline treated group, which might be attributed to the impeded drug permeation in poorly permeable tumor models 44. Tel only slightly improved the accumulation of GLPM at the tumor position. Notably, the GLPM/Tel treated group exhibited much better tumor mass accumulation than the saline and Tel treatments, which might be due to the prolonged circulation time and enhanced stability *in vivo* as well as the tumor mass specific delivery of Tel by GLPM. In addition, the distribution of GLPM also located in the liver as shown in the off-target signals as a result of positive charged surface 42. After 36h, tumor masses were excised and visualized as shown in Figure 4D, and the fluorescence in GLPM/Tel groups showed a higher distribution pattern compared with that in the saline and Tel treatment groups. For the semi-quantitative analysis, the fluorescence intensity of the tumor masses in the GLPM/Tel group was approximately 1.93- and 1.60- fold higher than in saline and Tel group, respectively.

To further investigate the change in intratumoral distribution after drug treatment, tumor masses were dissected and visualized by confocal microscopy. As shown in Figure 4E, the retention of GLPM was mainly prolonged in small areas and the distributed GLPM was not able to permeate deeper in the saline group. After Tel administration, more GLPM was delivered to the tumor tissue. However, the fluorescence still presented a limited and heterogeneous pattern. In contrast, the fluorescence exhibited deeper and more uniform intratumoral distribution pattern after GLPM/Tel treatment. Collectively, both the *in vitro* and *in vivo* results make us speculate that the inhibited secretion of stromal components could promote the depth and homogeneity of permeation, which enables tumor cells inside tumor masses to be more accessible to the subsequently administered drug, as shown in Figure 4F.

### The mechanism of improved intratumoral permeation *in vivo*

**Figure 5:**
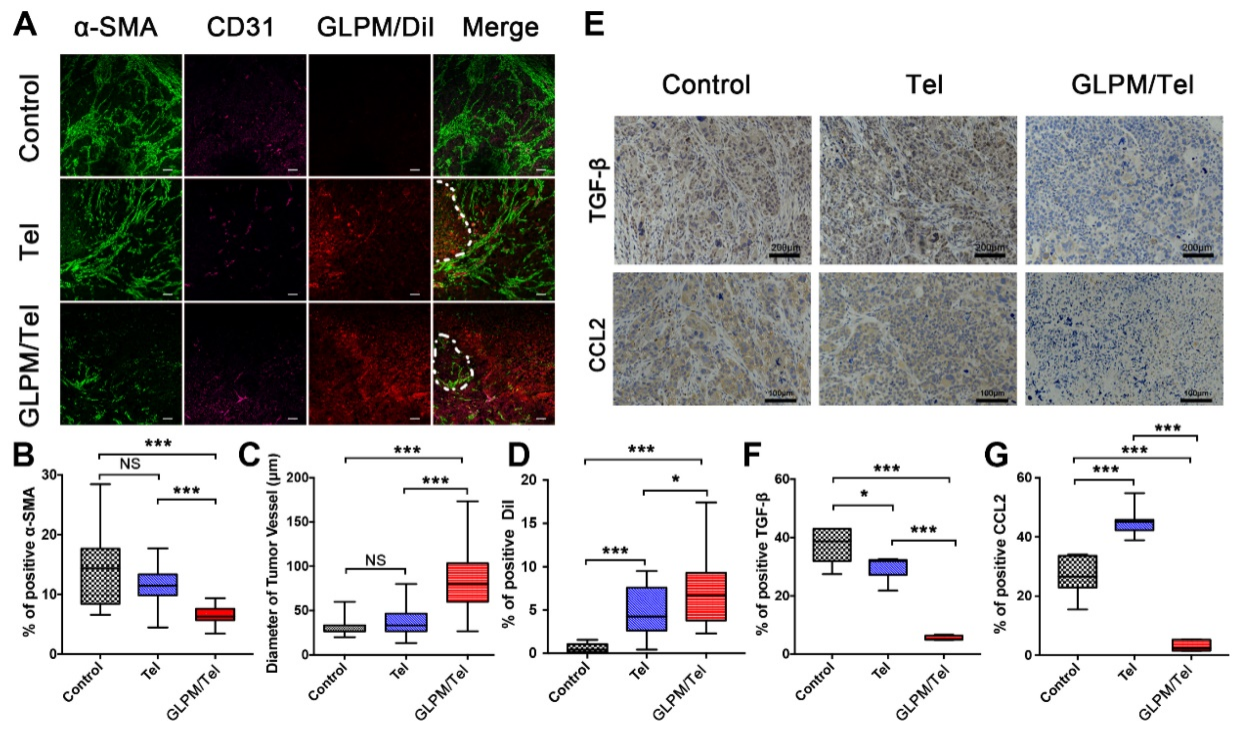
The remodeled tumor microenvironment at pre-administration of GLPM/Tel. A) Changes in the drug penetration capacity of breast tumors with saline, Tel and GLPM/Tel post-injection. Green fluorescence indicates *α*-SMA positive CAFs, and purple fluorescence indicates CD31 positive vessels, and red fluorescence indicates subsequently administered GLPM/DiI. Bar: 100μm. Semi-quantitative analysis of B) *α*-SMA positive CAFs, C) diameter of tumor vessels and D) penetrated DiI. E) Images of immunohistochemical staining and semi-quantitative analysis for F) TGF-*β* and G) CCL2 (n=5). *p < 0.05, **p < 0.01, ***p < 0.001 as determined by two-tailed Student's t-test.

After saline, Tel or GLPM/Tel pre-treatment, the hydrophobic fluorescence probe DiI loaded GLPM (GLPM/DiI) was subsequently administered to monitor the distribution patterns of micelles. Sections of the tumor masses were also stained to measure the expression and distribution patterns of CAFs and tumor vessels. As shown in Figure 5B, Tel and GLPM/Tel had various degrees of effectiveness on the inhibition of the activity of *α*-SMA positive CAFs *in vivo*. Almost 60% of CAFs were inhibited, and the well-organized CAFs were collapsed by GLPM/Tel. In contrast, less than 30% were inhibited by Tel, in which the physical barrier composed of CAFs and their secretions still existed (Figure 5A). The little cytotoxicity observed in activated NIH/3T3 cells by Tel and GLPM/Tel (Figure S10) indicated that the decreased amount of *α*-SMA was attributed to the decreased amount of CAFs rather than direct elimination.

An anti-angiogenic strategy was able to normalize the abnormal structure and function of the tumor vessels, enabling a more efficient intratumoral perfusion of blood and oxygen 45, 46. The anti-angiogenic efficacy of Tel and GLPM/Tel on HUVEC was confirmed above, and the effect on tumor vessels* in vivo* was also evaluated as shown in Figure 5C. The diameter of the tumor vessels increased significantly after GLPM/Tel treatment compared to the Tel and saline groups, which could improve the perfusion of decompressed vessels as a result of the pruned abnormal capillaries and inhibited stromal components inside the tumor masses. Notably, *α*-SMA and CD31 are negatively correlated as calculated by ImageJ software (Figure S15). The amount of distributed GLPM/DiI into tumor masses (Figure 5D) in the GLPM/Tel and Tel groups presented 11.9- and 7.3-fold increases, respectively, compared with the saline group. Notably, after the elimination of the stromal components by decreasing CAFs activity, GLPM/DiI was observed in areas with less green fluorescence (CAFs), as circled by dotted lines in Figure 5A and analyzed in Figure S16. Surprisingly, the increased tumor vessels seemed to only slightly improve drug distribution, which is attributed to the existing abundant and well-organized tumor stroma, as shown in Figure S17. Thus, these results collaboratively indicated that inhibition of the secretion of stroma components might complement the insufficient penetration in drug delivery to a considerable extent.

It has been demonstrated that the activation of the CAFs phenotype and CTGF are both TGF-*β* dependent 36. Moreover, the abundant TGF-*β* and a majority of the bioactive soluble factors secreted by CAFs, such as CCL2, could induce soluble factor-mediated drug resistance (SFM-DR) 47. Herein, the expression levels of representative TGF-*β* and CCL2* in vivo* were evaluated after drug treatments. As shown in Figure 5E, abundant TGF-*β* and CCL2 were observed in the saline group. Tel and GLPM/Tel at a concentration of 6 mg/kg strikingly reduced the area of TGF-*β* by 20.1% and 85.3% of the initial area, respectively, which could prohibit the activation of normal fibroblast cells to CAFs by TGF-*β.* Notably, there was nearly a 90% decrease in the area of CCL2 after GLPM/Tel administration, in contrast to the increased CCL2 area in the Tel group. In addition, the characteristic pathological structure (tumor clusters divided by stroma components) could be clearly observed in saline- and Tel-treated groups, which indicated a negligible effect on the compact and regular tumor stroma by Tel as a result of the short half-time and unspecific tumor distribution during blood circulation.

**Figure 6:**
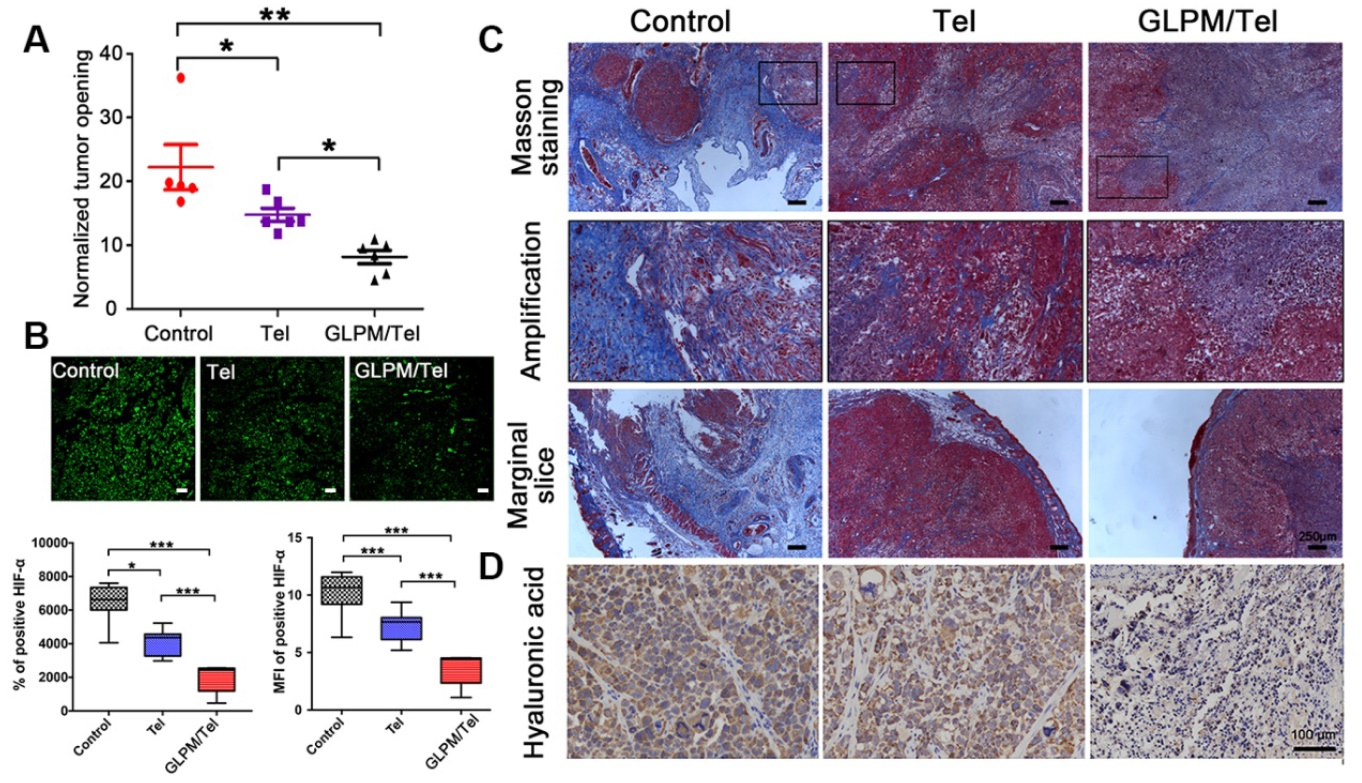
Investigation of alleviated solid stress at post-administration of GLPM/Tel. A) Estimation of solid stress in breast tumors surgically excised from mice after saline, Tel and GLPM/Tel treatment. B) Images and semi-quantitative analysis of HIF-α stained by immunofluorescent staining (n=5). Bar: 100μm. *p < 0.05, **p < 0.01, ***p < 0.001 as determined by two-tailed Student's t-test. C) Collagen stained by Masson staining. D) Hyaluronic acid staining by immunochemical staining was performed to investigate the remodeled tumor stroma after different drug treatments.

The solid components inside a tumor mass such as cancer, stroma cells, collagen and hyaluronic acid collaboratively contributed to physical forces (regarded as solid stress). Deletion of any or all of these components could alleviate solid stress to reopen compressed vessels, which could improve the perfusion and delivery of drugs 48, 49. Thus, it was reasonable to speculate that the decreased activity of CAFs by GLPM/Tel could alleviate the solid stress. As exhibited in Figure 6A, there was only a 21.2% decrease in solid stress after Tel treatment compared with saline, while GLPM/Tel exhibited superiority in decreasing the solid stress by 56.5%, which could account for the increased diameter of the tumor vessels. Aberrant vessels with poor blood flow could lead to hypoxic conditions, which promotes tumor progression, immunosuppression, inflammation, invasion and metastasis. After alleviating the solid stress by Tel and GLPM/Tel, the expression of HIF-*α* was evaluated by immunofluorescent staining as shown in Figure 6B. Consistent with our expectations, the expression of HIF-α was decreased by 28.2% and 64.5% after Tel and GLPM/Tel treatment, respectively, compared with after saline treatment, which could be attributed to the alleviated solid stress and decompressed vessels.

Total tissue pressure was strongly correlated with collagen area fraction across various tumor types and locations 50-52. However, the pressure was not directly influenced by the hyaluronic acid occupied within the tumor interstitium 53. Instead, collagen makes compressive stress to be applied to hyaluronic acid. In addition, hyaluronic acid was responsible for transmitting compressive stress to vessels. A strong correlation between the pressure and the ratio of hyaluronic acid to collagen area fractions was also confirmed 36. The compact ECM consisting of collagen and HA in tumors might also act as a physical obstacle, restricting drug penetration, in which case both HA and collagen are potential targets for improving antitumor therapies. Targeting the source of stromal components-CAFs with GLPM/Tel has great potential to achieve the purposed strategy of enhancing drug delivery, permeation and sensitization of cancer cells.

As shown in Figure 6C, masson staining was adopted to explore the changes in the content and distribution patterns of collagen after drug treatment. “Finger-like” collagen could be found all over the tumor mass, which defended cancer cells as “tough guards”. Moreover, evidence has demonstrated that elevated collagen area fractions in tumors can increase the irregularity and complexity of distribution patterns, which increases solid stress and decreased molecular uptake. Notably, a significant decrease in collagen was observed after Tel and GLPM/Tel treatments. In spite of this, the organized collagen structure could still be observed in the amplified images. Conversely, the amount and the structure of “finger-like” collagen, which can also result in cell adhesion mediated drug resistance (CAM-DR), was destroyed after GLPM/Tel treatment. In addition, the thickness of the peripheral collagen also shrank, which could decrease the tensile force to alleviate solid stress.

Collagen is not the only contributor to elevated solid stress. Thus, hyaluronic acid was further measured by immunohistochemical staining as shown in Figure 6D. As mentioned above, hyaluronic acid acts as a transporter of compressive stress to vessels, which means that the integrity and consistency are crucial for compressive stress transmission. Tel did not show an obvious effect on the hyaluronic acid distribution compared with saline. Notably, hyaluronic acid lacked plump conditions and integrity to exert transmitting stress functions *in vivo* after GLPM/Tel treatment. During the experiments, the tumor volumes were also monitored, as shown in Figure S18. The overlapping tumor growth curve of Tel with saline demonstrated a negligible effect on tumor growth. Surprisingly, GLPM/Tel exhibited impressive antitumor efficacy due to its inherent pharmacological activities and tumor microenvironment remodeling effect. The weight of excised tumor masses in Figure S19 confirmed the antitumor efficacy of GLPM/Tel. The weight of the mice in Figure S20 exhibited the good safety of Tel and GLPM/Tel. In summary, by virtue of the constructed GLPM, telmisartan was encapsulated for the specific delivery to tumor masses. Then, GLPM/Tel was trapped by the tumor stroma owing to its pathological structure in desmoplastic breast tumors. With decreasing the amount of CAFs, stromal components such as collagen, HA and bioactive cytokines were significantly affected. In terms of drug penetration, collagen along with hyaluronic acid was inhibited so that the solid stress was alleviated. Consequently, tumor vessels were decompressed and pruned to restore perfusion functions for improving drug delivery, in which case more drug could be delivered to tumor masses. Afterwards, access of the tumor cells inside was improved by subsequently administered drugs along with improved intratumoral drug penetration.

### Antitumor therapy evaluation of the rationally designed combination of GLPM/Tel and GLPM/DOX *in vivo*

**Figure 7:**
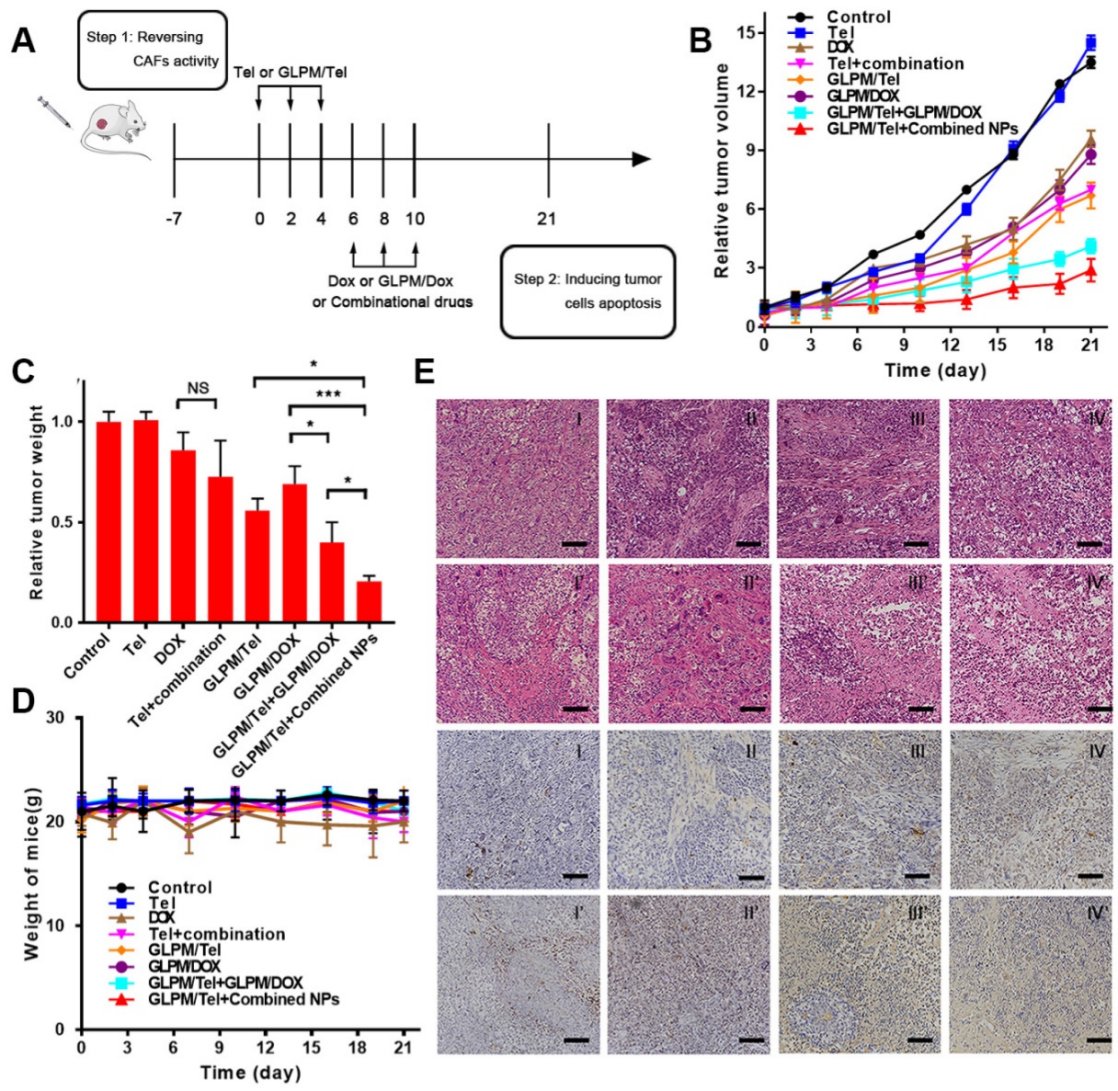
Antitumor efficacy of combination administration strategy. a) Scheme of the strategy for combinational therapies* in vivo*. b) *In vivo* tumor inhibition evaluation of different drug treatments. The results are shown as the means ± SD. c) The weights of excised tumor masses and d) the weight of mice was monitored during drug treatment (n=5). *p < 0.05, **p < 0.01, ***p < 0.001 as determined by two-tailed Student's t-test. e) HE staining and TUNEL staining for tumor mass were adopted after different drugs treatments to study the efficacy of remodelingthe tumor microenvironment and subsequent synergistic antitumor efficacy. Scale bar: 100*μ*m. I: saline, II: Tel, III: DOX, IV: Tel+combination (Tel+DOX); I': GLPM/Tel, II': GLPM/DOX, III': GLPM/Tel+GLPM/DOX, IV': GLPM/Tel+combined NPs (GLPM/Tel+GLPM/DOX).

The efficacy of decreasing *α*-SMA positive CAFs by GLPM/Tel *in vitro* and on breast xenografts was verified as above. The synergetic antitumor efficacy of GLPM/Tel with GLPM/DOX has been demonstrated as shown in Figure 3. In addition, not only is the identity of the individual drugs important, but the order in which they are administered can also play a crucial role 54. Thus, the antitumor efficacy of the rationally designed combination of GLPM/Tel and GLPM/DOX was explored on MCF-7 xenograft tumors. As shown in Figure 7B, Tel showed little effect on tumor growth. As expected, DOX achieved only minor antitumor efficacy at the dose of 3 mg/kg, which is much less than the dose normally used 55, 56. The inhibition efficacy of the combined Tel and DOX did not show any significant difference compared to DOX treatment owing to the lack of tumor mass-targeting and instability *in vivo*. GLPM/DOX substantially arrested tumor growth as expected. To verify the synergetic antitumor effect of GLPM/Tel and GLPM/DOX, two dosing regimens were carried out. At the end of GLPM/Tel administration, subsequent GLPM/DOX at a final dose of 3 mg/kg or combined GLPM/Tel and GLPM/DOX (equivalent dosage of DOX at molar ratio of 2:1) was administered three times. In accordance with the results in MCTSs, both groups exhibited remarkable suppression of tumor growth, yielding statistically improved therapeutic outcomes compared to only GLPM/DOX treatment. Notably, subsequent administration of the combined NPs presented a preferable therapeutic efficacy than other groups with the highest inhibitory ratio of approximately 78.5%. This could be attributed to the enhanced drug accumulation and penetration as well as the synergistic antitumor effects of subsequently administered GLPM/DOX and GLPM/Tel in MCF-7 cells. At the end of tumor volume monitoring, tumor masses were dissected and weighed as shown in Figure 7C, which was consistent with the data of the tumor volume *in vivo*. As an indicator of the *in vivo* safety and biocompatibility for the drugs, the weight of tumor bearing nude mice was monitored as shown in Figure 7D. No obvious weight was decreased except for in the DOX and combined Tel and DOX groups due to the known cardiotoxicity 28. The hematoxylin and eosin stain (HE) method for major organs was adopted to evaluate the *in vivo* safety after drug treatments. As shown in Figure S21, DOX as well as combined Tel and DOX treated mice both showed cardiotoxicity. In contrast, the other groups did not exhibit any significant pathological abnormalities.

A large amount of existing tumor stroma in the saline group presented compact and regular structures, which act as obstacles for drug penetration, as shown in Figure 7E. The same tendency in the Tel groups indicated an insufficient effect. Consequently, the thick and organized tumor stroma, which could severely trap the administered DOX to produce off-target effects, reminded us that neglecting tumor stroma targeting in a normally adopted antitumor strategy needed to be re-estimated. Less compact structure of tumor section, vacuole condition and irregular shape tumor cells were visualized after DOX and combined Tel and DOX treatments. In virtue of the constructed GLPM, GLPM/DOX exhibited equivalent antitumor efficacy to DOX. However, the “finger-like” barrier still existed, and only a small vacancy existed in the tumor cells as a result of the dense tumor stroma. After treatment with GLPM/Tel, the structure of the tumor stroma became less compact so that the tumor cells inside could be exposed to further internalization of the subsequently injected drugs. It is worth noting that enhanced antitumor efficacy, collapsed stromal structure, scattered apoptotic cells and destroyed tumor masses were observed in both combinational nano-carrier based antitumor therapy groups. The results demonstrated the superiority and necessity of intervening with the tumor stroma. Although subsequent administration of GLPM/DOX improved the antitumor effect after GLPM/Tel treatment, a few tumor clusters remained. In contrast, in addition to the elimination of tumor stroma by GLPM/Tel, the subsequent administration of the combined GLPM/Tel and GLPM/DOX at a molar ratio of 2:1 prompted tumor cell to become apoptotic, and almost all tumor masses were destroyed.

TUNEL staining was also utilized to further confirm the potential of the purposed antitumor strategy. As demonstrated, less apoptotic cells could be observed in the slices of saline-, Tel- and GLPM/Tel-treated groups, which could indicate that the elimination of tumor-promoting stromal components was the main reason for the achieved antitumor effect of GLPM/Tel. Although majority of the tumor cells were eliminated, a few tumor clusters still existed in the sequential administration groups of GLPM/Tel and GLPM/DOX. Additionally, few tumor cells remained in the primary shape and condition, and only apoptotic cells as well as cellular debris in the GLPM/Tel+combined NPs group could be observed. Overall, GLPM/Tel exhibited great potential to inhibit tumor growth by remodeling the tumor microenvironment and synergistically exerting antitumor efficiency with GLPM/DOX.

## Conclusion

Recently, the interaction between tumor cells and stromal cells such as CAFs attracted much attention for reversing tumor microenvironment induced drug resistance and enhancing drugs distribution 57-62. However, rare study concentrated on the influence of pathological structure on the depth and homogeneity of penetrated drugs in desmoplastic tumors. Thus, sequential combinational dosing strategies are put forward according to the specific pathological structure. In summary, well-defined and simply constructed glycolipid polymeric micelles encapsulating an angiotensin II receptor I inhibitor were prepared.

Pre-administered GLPM/Tel sufficiently decreased CAFs derived stromal components, such as collagen and hyaluronic acid, alleviated solid stress and reduced hypoxia in tumor masses. Together with pruning abnormal capillaries, the capacity of blood perfusion was jointly restored, which paved the way for the deeper and more uniform penetration of subsequently administered drugs inside tumor masses. In addition, CAFs secreted cytokines, such as TGF-*β* and CCL2, were inhibited along with the decreased activity of CAFs, which in turn further decreased the activity of CAFs. Afterwards, the subsequent administration of combined GLPM/Tel and GLPM/DOX at a molar ratio of 2:1 continued to decrease the secretion of the CAFs-derived stroma, and achieved synergistic antitumor efficacy through the PPAR-*γ* signal pathway. In brief, the primary and suggestive results provide us the potential strategy to exploit the weaknesses of the interaction between the tumor stroma and cancer cells by time-staggered administrations, which could promote subsequently administered combinational therapies beyond the conventional tumor cells only targeted combinational therapies.

## Supplementary Material

Supplementary figures and tables.

## Figures and Tables

**Table 1:** Characteristics of micelles.

Micelles	Dn (nm)	Zeta potentials (mV)	DL (%)	EE (%)	PDI
GLPM	138.59±18.68	28.7±1.2			0.09±0.01
GLPM/DOX	110.65±3.46	23.9±2.0	8.15	88.2	0.12±0.02
GLPM/Tel	76.32±4.30	26.6±0.7	8.06	87.6	0.16±0.03

Data represent the mean ± standard deviation (n=3). PDI: polydispersity index
