# Supplementary material for: Reversing activity of cancer associated fibroblast for staged glycolipid micelles against internal breast tumor cells
Source: Theranostics. 2019 Sep 19;9(23):6764–79. doi: 10.7150/thno.36334 (PMC6815968; doi:10.7150/thno.36334)
Supplement: Supplementary file 1 — Supplementary figures and tables. [file thnov09p6764s1.pdf]

Supporting information

Reversing activity of cancer associated fibroblast for staged glycolipid micelles against internal breast tumor cells

Yun Zhu,† Fangying Yu,‡ Yanan Tan,† Yun Hong,§ Tingting Meng,‡ Yupeng Liu,‡ Suhuan Dai,‡ Guoxi Qiu,‡ Hong Yuan,‡ and Fuqiang Hu*†‡

‡College of Pharmaceutical Science, Zhejiang University, 866 Yuhangtang Road, Hangzhou 310058, People's Republic of China.

† Ocean College, Zhejiang University, 1 Zheda Road, Zhoushan 316021, People's Republic of China.

§ The First Affiliated Hospital, College of Medicine, Zhejiang University, 79 Qingchun Road, Hangzhou 310058, China

*Corresponding Author: Prof. Fuqiang Hu

Tel/Fax: +86-571-88208439

E-mail: hufq@zju.edu.cn

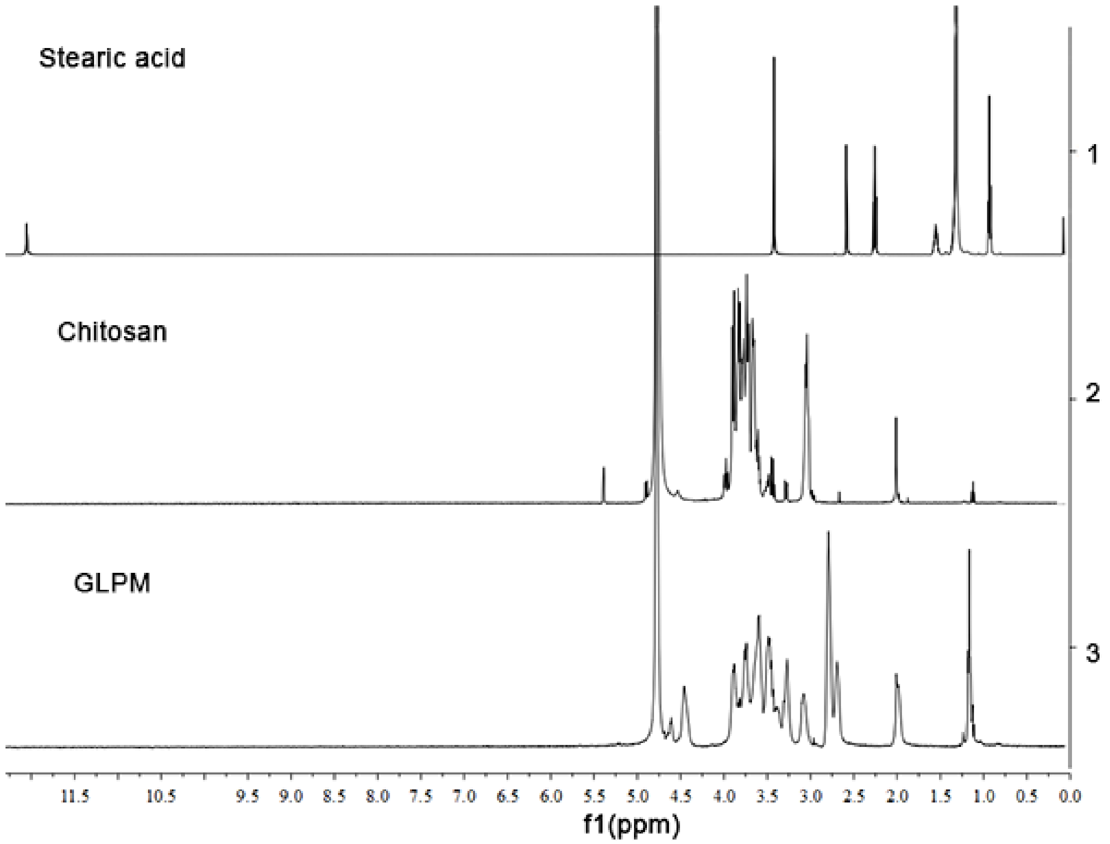

Figure S1. The 1H NMR spectroscopy of synthesized glycolipid polymer.

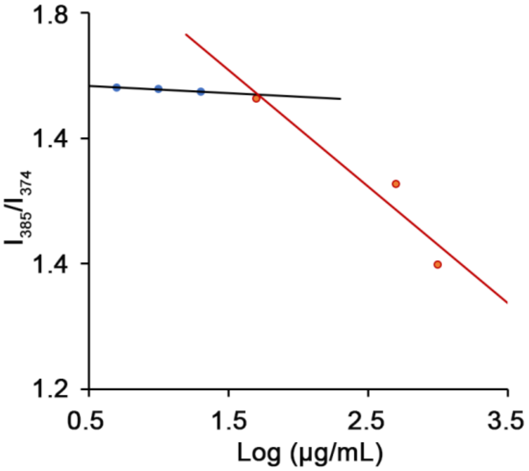

Figure S2. CMC value of GLPM in aqueous medium.

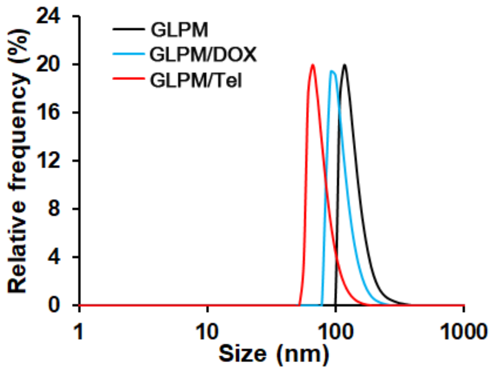

Figure S3. The size of GLPM, GLPM/DOX and GLPM/Tel measured by DLS.

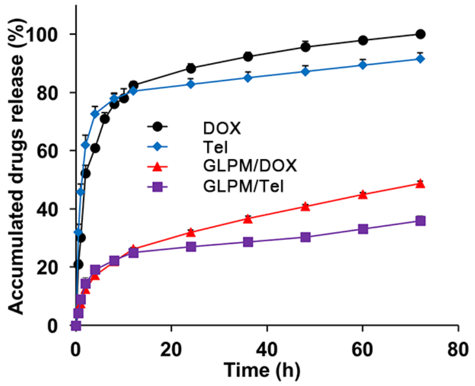

Figure S4. Drug release profiles of Tel, DOX, GLPM/Tel and GLPM/DOX after incubation with pH 7.4 PBS (10% fetal bovine serum).

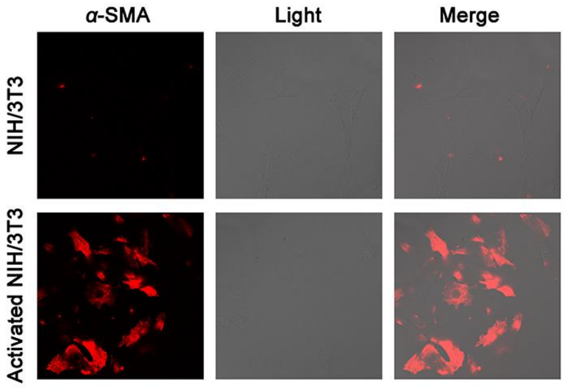

Figure S5. The activation of normal NIH/3T3 cells by supernatant collected from MCF-7 cells.

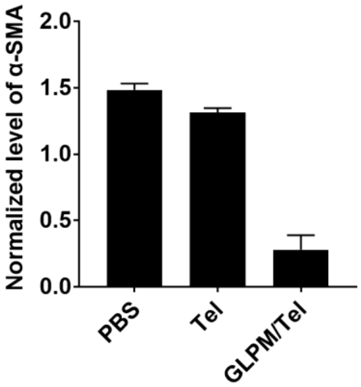

Figure S6. Semi-quantitative analysis of expressed α-SMA in activated NIH/3T3 cells after different drugs treatment.

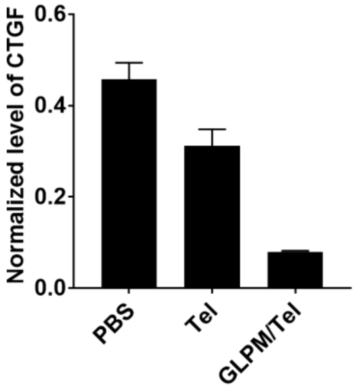

Figure S7. Semi-quantitative analysis of expressed CTGF in activated NIH/3T3 cells after different drugs treatment.

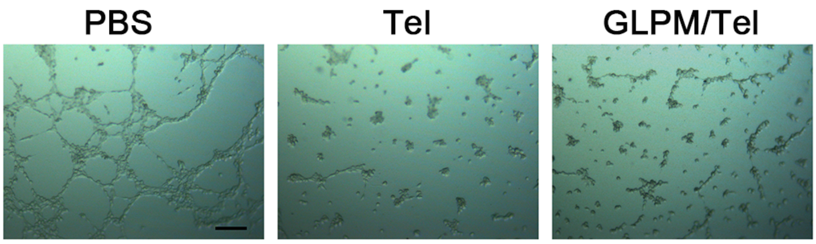

Figure S8. The anti-angiogenic efficacy of Tel and GLPM/Tel on HUVEC cell line *in vitro*.

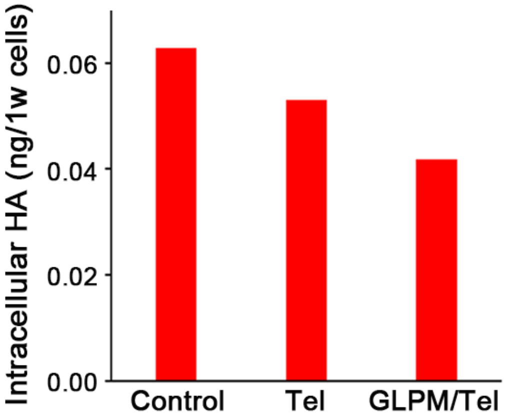

Figure S9. Quantitative analysis of intracellular HA after drugs treatment for 48h.

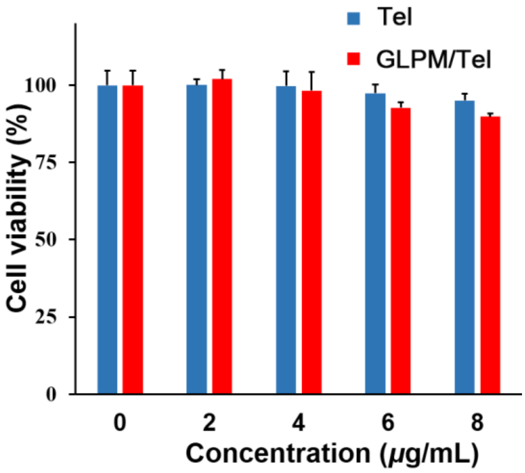

Figure S10. Cytotoxicity evaluation of Tel and GLPM/Tel on activated NIH/3T3 cell.

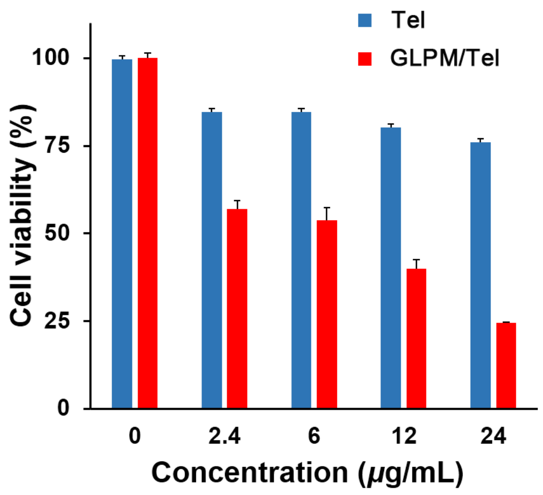

Figure S11. Cytotoxicity evaluation of Tel and GLPM/Tel on MCF-7 cell.

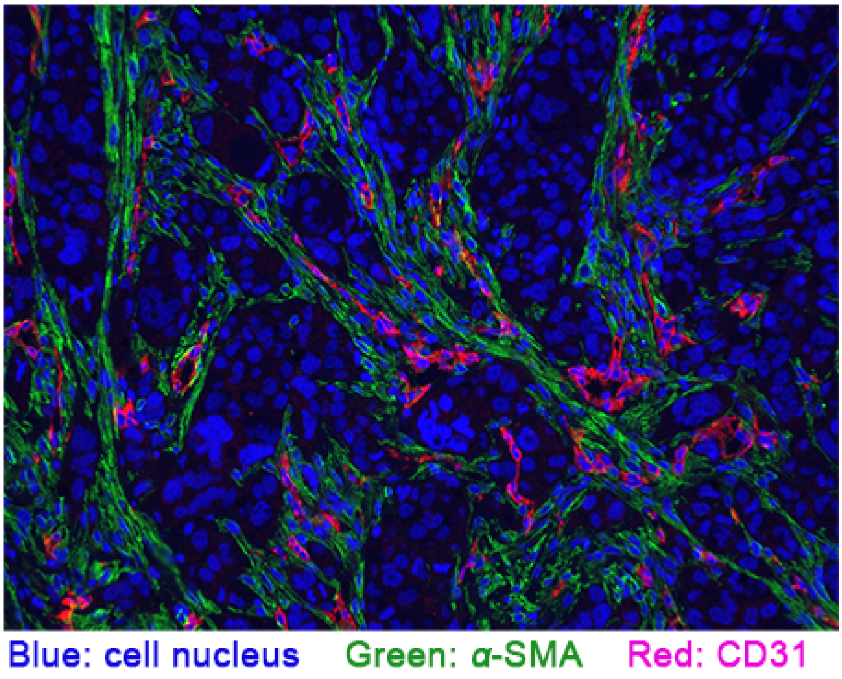

Figure S12. Immunofluorescent staining for the pathological structure on MCF-7 tumor mass.

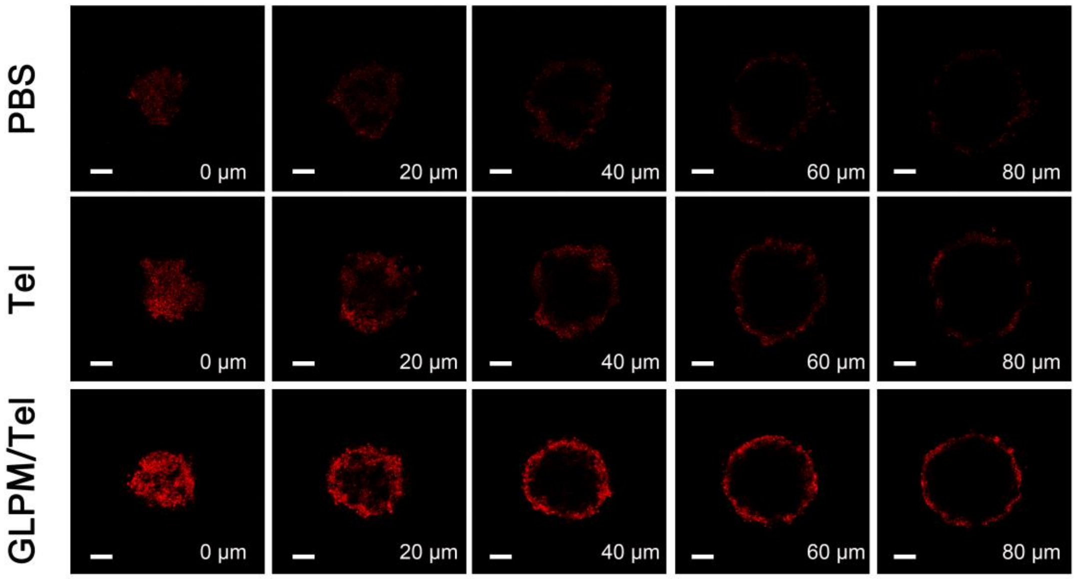

Figure S13. Z-stack images of penetration of GLPM/DOX in MCTSs composed of 4T1 cells and activated NIH/3T3 cells were visualized by confocal microscopy after PBS, Tel and GLPM/Tel treatment for 2 days. Bars: 100 μm.

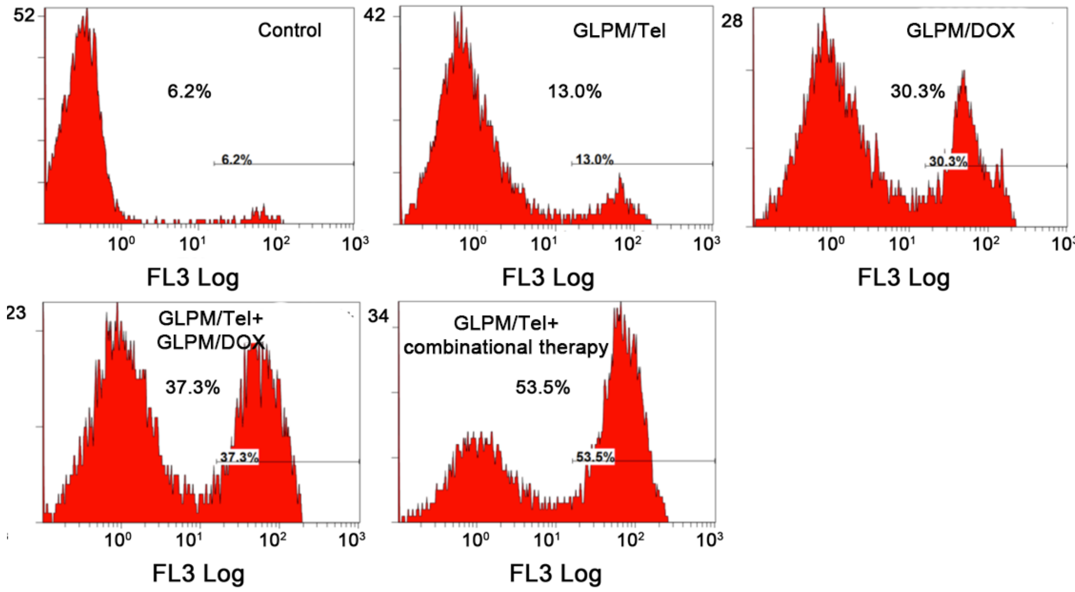

Figure S14. Antitumor efficacy of different drugs on MCTSs measured by PI staining.

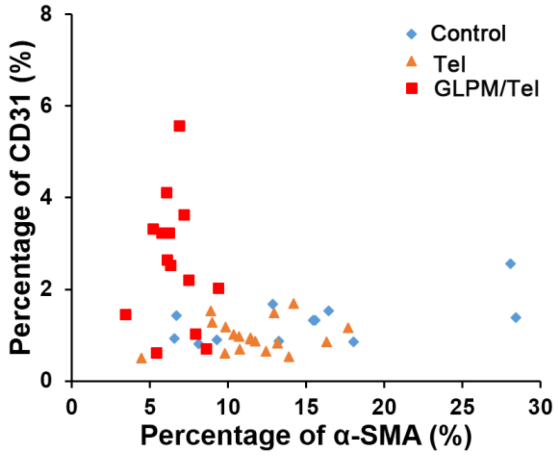

Figure S15. The correlation between *α*-SMA and CD31 calculated by imageJ software.

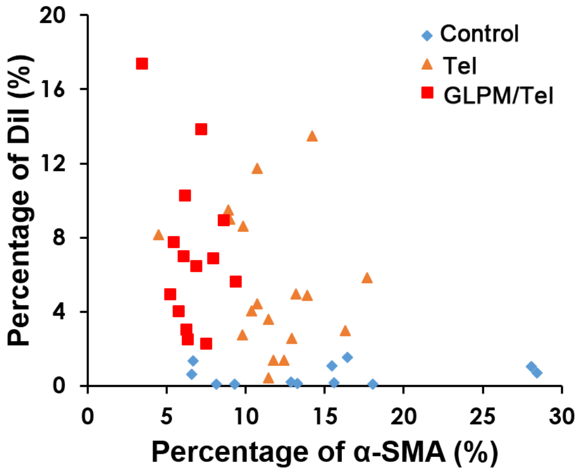

Figure S16. The correlation between *α*-SMA and DiI calculated by imageJ software.

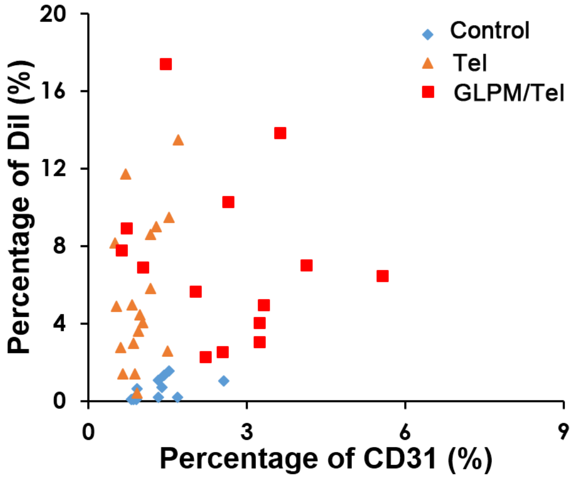

Figure S17. The correlation between CD31 and DiI calculated by imageJ software.

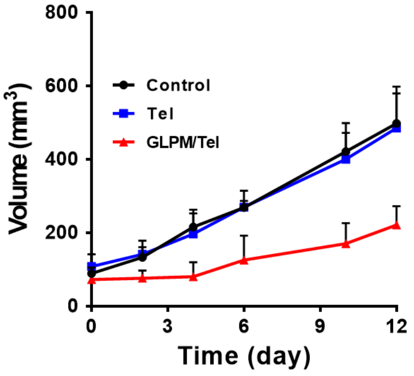

Figure S18. The volumes of MCF-7 tumor during saline, Tel and GLPM/Tel treatments.

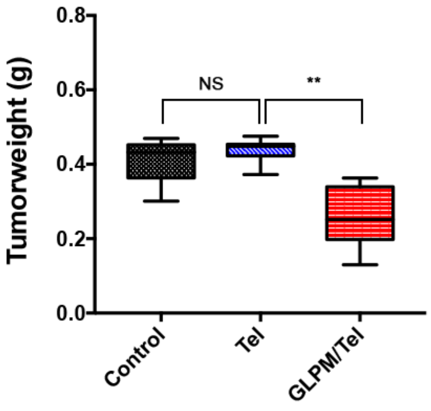

Figure S19. The weight of MCF-7 tumor masses after saline, Tel and GLPM/Tel treatment for three times. $^{**}p < 0.01$, $^{***}p < 0.001$ as determined by two-tailed student's t-test.

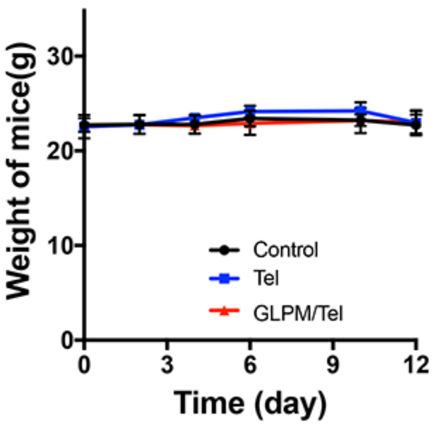

Figure S20. The weight of mice during drugs treatment.

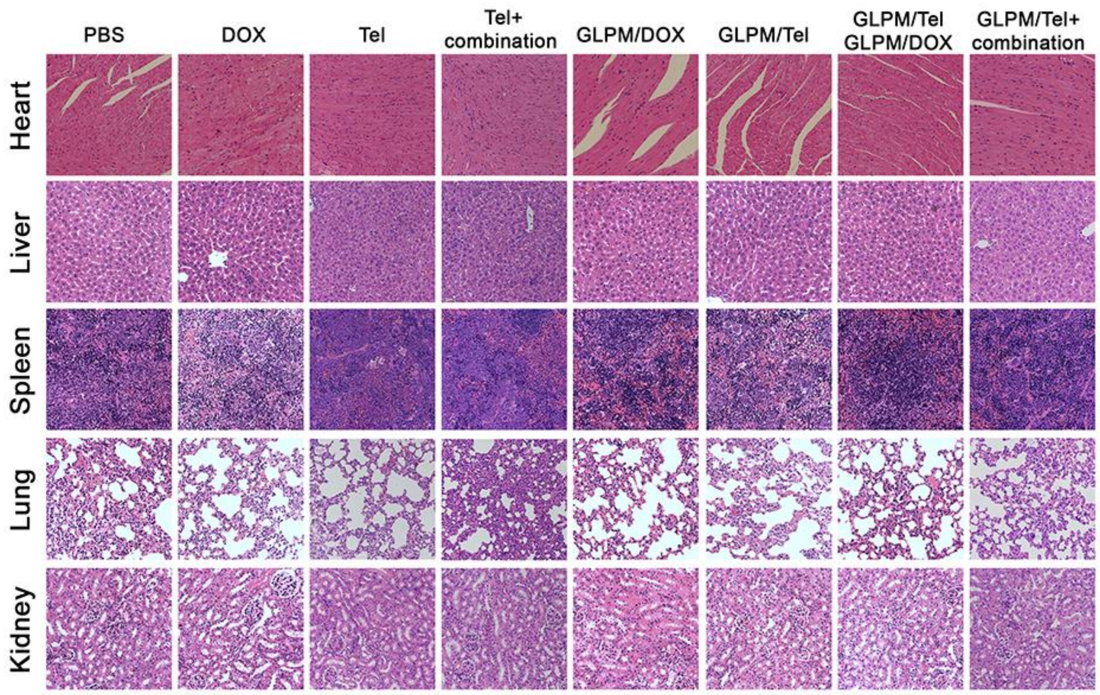

Figure S21. HE staining for major organs after drugs treatment.

Table S1. Depth of MCTSs after drugs treatment.

	**PBS**	**Tel**	**GLPM/Tel**	**GLPM/DOX**	**GLPM/Tel+ GLPM/DOX**	**GLPM/Tel+ combinational therapy**
**Penetration Depth**	70μm	140μm	140μm	/	/	/
**Apoptosis ratio**	6.2%	/	13.0%	30.3%	37.3%	53.5%
